# Supplementary material for: Sex-based differences in clearance of chronic Plasmodium falciparum infection
Source: eLife. 2020 Oct 27;9:e59872. doi: 10.7554/eLife.59872 (PMC7591246; doi:10.7554/eLife.59872)
Supplement: Supplementary file 1. [file elife-59872-supp1.docx]

**Supplemental File**

**Table of Contents**

1. File 1a: AMA-1 hemi-nested PCR protocol for amplicon deep-sequencing….….pages 3, 4
2. File 1b: Bioinformatics workflow ……………………………………………………………………………..….5
3. File 1c: Declining qPCR density over time in the cohort……………………………………..………..6
4. File 1d: Detailed explanation of skip rule criteria……….…………………………………………………7
5. File 1e: Haplotype sequences and frequencies…………………………………………….……………...8
6. File 1f: Sensitivity analysis of force of infection…………………………………………….……………..9
7. File 1g: Sensitivity analysis of clearance of infection…………..………………………………….10,11

**Supplemental File 1a. AMA-1 hemi-nested PCR protocol for amplicon deep-sequencing**

| **Primer** | **Sequence (5’→3’)** |
| --- | --- |
| Ama1OF | GCTGAAGTAGCTGGAACTCAA |
| Ama1R | TTTCCTGCATGTCTTGAACA |
| Ama1F+a* | acactctttccctacacgacgctcttccgatct-CCATCAGGGAAATGTCCAGT |
| Ama1R+a | gtgactggagttcagacgtgtgctcttccgatct-TTTCCTGCATGTCTTGAACA |

*a = adapter

**AMA1 Nested PCR Protocol**

25µl Reaction

| **Reagent** | **Initial Concentration** | **Volume Added (µl)** | **Final Concentration** |
| --- | --- | --- | --- |
| FastStart High Fidelity Buffer | 10X with 18mM MgCl2 | 2.5 | 1X (1.8mM MgCl2) |
| dNTPs | 10mM | 0.5 | 200uM each dNTP |
| Roche High Fidelity Taq | 5U / µl | 0.25 | 2.5 U |
| Pf AMA1OF primer | 10uM | 0.5 | 167nM |
| Pf AMA1R primer | 10uM | 0.5 | 167nM |
| Template DNA | - | 5 | - |
| Water | - | 15.75 | - |

Use same master mix protocol for Round 1 and Round 2, substituting “AMA1OF Primer” with “AMA1F-a”, “AMA1R primer” with “AMA1R-a primer” and “Template DNA” with “Round 1 PCR Product” in Round 2.

**Cycling Conditions**

1^st^ round PCR

| **Cycling Conditions R1** | **Temperature** | **Time** |
| --- | --- | --- |
| Step 1 | 95 C | 2 min |
| Step 2 | 95 C | 30 sec |
| Step 3 | 55 C | 30 sec |
| Step 4 | 72 C | 1 min |
| Step 5 | Repeat step 2-4 total 20X |  |
| Step 6 | 72 C | 10 min |
| Step 7 | 4 C or 12 C | forever |

2^nd^ round PCR

| **Cycling Conditions R2** | **Temperature** | **Time** |
| --- | --- | --- |
| Step 1 | 95 C | 2 min |
| Step 2 | 95 C | 30 sec |
| Step 3 | 55 C | 30 sec |
| Step 4 | 72 C | 1 min |
| Step 5 | Repeat step 2-4 total 10X, 20X, or 25X* |  |
| Step 6 | 72 C | 10 min |
| Step 7 | 4 C or 12 C | forever |

*10X for samples with parasite density between 10,000 and 1,000 parasites/µL
 20X for samples with parasite density between 1,000 and 10 parasites/µL
 25X for samples with parasite density between 10 and 0.1 parasites/µL

**INDEXING PCR**

50µL reaction

| **Reagent** | **Volume Added (ul)** |
| --- | --- |
| NEBNext MasterMix | 25 µL |
| Sample (2^nd^ round PCR product) | 15 µL |
| Barcode | 10 µL |

| **Temperature** | **Time** | **Cycles** |
| --- | --- | --- |
| 98 C | 30 sec |  |
| 98 C | 10 sec | 8-22 cycles |
| 65 C | 75 sec |  |
| 65 C | 5 min |  |
| 10 C | forever |  |

**Supplemental File 1b: Bioinformatics workflow**

**
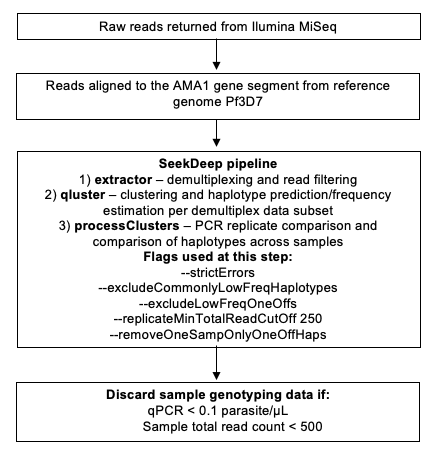
**

**Supplemental File 1c. Declining qPCR density over time in the cohort.**

**Supplemental File 1d. Detailed explanation of skip rule criteria**

| **By clone:**   1. “Skips” = dates on which haplotypes recovered from a genotyped sample did not include a clone seen previously in that individual, or where qPCR was negative 2. 4 skips in a row were required to call the infection with that clone cleared 3. If there were <= 3 skips and the same haplotype is detected again, infection with that clone is presumed to have continued throughout the dates of the skips 4. If there were 4 skips and the same haplotype was detected again, it was considered a new infection with that clone |
| --- |
| **By infection event:**   1. “Skips” = dates on which haplotypes recovered from a genotyped sample did not include any of the haplotypes included in an infection event, or where qPCR was negative 2. 4 skips in a row were required to call the infection event cleared 3. If there were <= 3 skips and any haplotype from the same infection event was detected again, that infection event is presumed to have continued throughout the dates of the skips 4. If there were 4 skips and haplotypes from a previous infection event were detected again, it was considered a new infection event |

**5. Supplemental File 1e. Haplotype sequences and frequencies**

**Read Percent:** total number of reads that are associated with the haplotype across all samples divided by the total number of reads in the experiment, multiplied by 100

**Sample Percent:** number of samples the haplotype was found in divided by the number of samples in the experiment (sums to greater than 100%, since multiple haplotypes can be found in same sample), multiplied by 100

**Haplotype Percent:** sum of the number of times a haplotype was recovered over total number of haplotypes recovered, multiplied by 100

**Supplemental File 1f. Sensitivity analysis of molecular force of infection: Table 2 replicated using 2 skips or 1 skip**

**Molecular force of infection (FOI) by clone and by infection event, stratified by age and sex: calculated using 2 skips**

| **Molecular force of infection (FOI)** | **Sex** | **Age category** | | | |
| --- | --- | --- | --- | --- | --- |
|  |  | **All** | **< 5 years** | **5-15 years** | **16 years or older** |
| By clone, ppy* (95% CI) | All | 0.21 (0.16-0.29) | 0.15 (0.07-0.28) | 0.23 (0.10-0.52) | 0.24 (0.11-0.55) |
|  | Male | 0.23 (0.15-0.36) | 0.17 (0.07-0.40) | 0.24 (0.12-0.48) | 0.27 (0.12-0.60) |
|  | Female | 0.19 (0.11-0.35) | 0.12 (0.03-0.47) | 0.22 (0.09-0.51) | 0.23 (0.08-0.61) |
| By event, ppy* (95% CI) | All | 0.18 (0.14-0.23) | 0.11 (0.06-0.18) | 0.20 (0.11-0.39) | 0.20 (0.10-0.39) |
|  | Male | 0.20 (0.14-0.26) | 0.16 (0.08-0.30) | 0.21 (0.13-0.35) | 0.20 (0.11-0.37) |
|  | Female | 0.16 (0.10-0.26) | 0.06 (0.02-0.17) | 0.20 (0.10-0.38) | 0.20 (0.09-0.45) |

*per person-year

**Molecular force of infection (FOI) by clone and by infection event, stratified by age and sex: calculated using 1 skip**

| **Molecular force of infection (FOI)** | **Sex** | **Age category** | | | |
| --- | --- | --- | --- | --- | --- |
|  |  | **All** | **< 5 years** | **5-15 years** | **16 years or older** |
| By clone, ppy* (95% CI) | All | 0.32 (0.23-0.42) | 0.16 (0.08-0.30) | 0.44 (0.20-0.94) | 0.29 (0.13-0.64) |
|  | Male | 0.38 (0.24-0.59) | 0.19 (0.08-0.42) | 0.50 (0.27-0.93) | 0.35 (0.17-0.73) |
|  | Female | 0.26 (0.15-0.45) | 0.13 (0.04-0.49) | 0.36 (0.16-0.78) | 0.25 (0.10-0.64) |
| By event, ppy* (95% CI) | All | 0.28 (0.22-0.37) | 0.12 (0.07-0.20) | 0.38 (0.20-0.72) | 0.30 (0.16-0.56) |
|  | Male | 0.33 (0.23-0.48) | 0.17 (0.09-0.33) | 0.42 (0.24-0.73) | 0.32 (0.18-0.58) |
|  | Female | 0.24 (0.15-0.39) | 0.07 (0.03-0.20) | 0.33 (0.16-0.67) | 0.28 (0.13-0.60) |

*per person-year

**Supplemental File 1g. Sensitivity analysis of duration of infection: Table 3 replicated using 2 skips or 1 skip**

**Hazard ratios for rates of clearance of infection, by clone and by infection event: calculated using 2 skips**

| **Predictors** | **Categories** | **Hazard ratio by clone (95% CI)** | | **Hazard ratio by infection event (95% CI)** | |
| --- | --- | --- | --- | --- | --- |
|  |  | **Unadjusted** | **Adjusted** | **Unadjusted** | **Adjusted** |
| Sex | Male | ref | ref | ref | ref |
|  | Female | 2.05 (1.29-3.26) | 1.92 (1.33 – 2.78) | 2.30 (1.35 – 3.90) | 2.24 (1.49 – 3.37) |
| Age | 16 years or greater | ref | ref | ref | ref |
|  | 5-15 years | 0.48 (0.30 – 0.78) | 0.62 (0.40 – 0.96) | 0.55 (0.31 – 0.98) | 0.83 (0.53 – 1.31) |
|  | < 5 years | 1.65 (0.88 – 3.08) | 1.83 (1.04 – 3.23) | 2.00 (1.02 – 3.90) | 1.71 (1.02 – 2.87) |
| Complexity of infection (COI) | Polyclonal (COI > 1) | ref | -- | ref | -- |
|  | Monoclonal (COI = 1) | 1.60 (1.04 – 2.47) | -- | 0.97 (0.52 – 1.79) | -- |
| Infection status | Present at baseline | ref | ref | ref | ref |
|  | New infection | 1.49 (0.99 – 2.24) | 1.27 (0.85 – 1.88) | 3.19 (2.01 – 5.07) | 2.68 (1.80 – 4.00) |
| Parasite density * | | 0.72 (0.60 – 0.88) | 0.71 (0.59 – 0.86) | 0.46 (0.38 - 0.54) | 0.50 (0.42 – 0.60) |

*Increasing parasite density (log10) in parasites/microliter, as measured by qPCR

**Hazard ratios for rates of clearance of infection, by clone and by infection event: calculated using 1 skip**

| **Predictors** | **Categories** | **Hazard ratio by clone (95% CI)** | | **Hazard ratio by infection event (95% CI)** | |
| --- | --- | --- | --- | --- | --- |
|  |  | **Unadjusted** | **Adjusted** | **Unadjusted** | **Adjusted** |
| Sex | Male | ref | ref | ref | ref |
|  | Female | 1.64 (1.12-2.39) | 1.59 (1.12 – 2.27) | 1.84 (1.28 – 2.63) | 1.70 (1.18 – 2.46) |
| Age | 16 years or greater | ref | ref | ref | ref |
|  | 5-15 years | 0.85 (0.55 – 1.32) | 0.90 (0.59 – 1.36) | 0.76 (0.51 – 1.13) | 1.03 (0.69 – 1.55) |
|  | < 5 years | 1.49 (0.76 – 2.90) | 1.63 (0.85 – 3.14) | 1.36 (0.76 – 2.42) | 1.41 (0.79 – 2.52) |
| Complexity of infection (COI) | Polyclonal (COI > 1) | ref | -- | ref | -- |
|  | Monoclonal (COI = 1) | 1.48 (1.02 – 2.17) | -- | 1.20 (0.82 – 1.75) | -- |
| Infection status | Present at baseline | ref | ref | ref | ref |
|  | New infection | 1.00 (0.69 – 1.44) | 1.04 (0.73 – 1.50) | 2.63 (1.74 – 3.99) | 2.71 (1.83 – 4.01) |
| Parasite density * | | 0.81 (0.67 – 0.99) | 0.80 (0.66 – 0.96) | 0.63 (0.54 – 0.74) | 0.65 (0.56 – 0.75) |

*Increasing parasite density (log10) in parasites/microliter, as measured by qPCR
